# Calcium Disorders in Rheumatic Diseases: An Overlooked Problem With Major Clinical Implications

**DOI:** 10.7759/cureus.106305

**Published:** 2026-04-01

**Authors:** Balakrishnan Navaneethakrishnan, Gopalakrishnan Syamala Nikhila

**Affiliations:** 1 Department of Rheumatology, Avinash Hospitals, Chennai, IND; 2 Department of Rheumatology, Gleneagles HealthCity, Chennai, IND

**Keywords:** calcium disorders, crystal deposition, inflammation, rheumatic diseases, vitamin d

## Abstract

Rheumatoid arthritis (RA), systemic lupus erythematosus (SLE), spondyloarthritis, calcium pyrophosphate deposition disease, and the therapies used to treat them, particularly disease-modifying antirheumatic drugs, exemplify clinical contexts where calcium homeostasis has underappreciated relevance. Calcium balance is central to skeletal integrity, immune signalling, and multisystem function in rheumatic diseases, yet it is frequently overshadowed by inflammatory priorities. Despite growing evidence linking calcium disorders to morbidity, integration into routine rheumatology care remains limited, representing a persistent clinical gap. The objective of this review is to synthesize current knowledge on the spectrum, mechanisms, manifestations, and management of calcium disorders in rheumatic diseases. A narrative review was conducted using PubMed, Scopus, and Web of Science, covering literature published between 2015 and 2025, including original studies and reviews. Chronic inflammation, therapeutic exposures, endocrine disruption, and renal involvement collectively drive hypocalcemia, hypercalcemia, secondary hyperparathyroidism, and crystal deposition disorders. These abnormalities contribute to skeletal fragility, renal complications, cardiovascular calcification, and diagnostic uncertainty. Integrating calcium assessment into rheumatologic practice has important implications for risk stratification, individualized therapy, and the prevention of long-term complications. Calcium disorders should be recognized as integral components of rheumatic disease biology rather than incidental comorbidities. The systematic, multidisciplinary integration of calcium homeostasis into rheumatology care can meaningfully improve patient outcomes.

## Introduction and background

Calcium homeostasis is a fundamental physiological process essential for musculoskeletal integrity, neuromuscular excitability, intracellular signalling, and immune control, making it a critical component of overall systemic health that is often underrecognized in clinical practice [1]. Historically, disturbances of calcium balance in clinical practice have been primarily associated with endocrine or renal illnesses, and their relevance in rheumatic diseases has been comparatively less explored [2]. However, growing evidence suggests that calcium disorders are frequent and clinically relevant complications of a wide spectrum of inflammatory and autoimmune rheumatic diseases [3]. Rheumatoid arthritis (RA), systemic lupus erythematosus (SLE), spondyloarthritis, systemic sclerosis, and crystal-induced arthropathies are examples of chronic inflammatory rheumatic diseases that significantly impact calcium metabolism due to interrelated inflammatory, hormonal, renal, and skeletal mechanisms [4]. Persistent systemic inflammation leads to bone remodelling that amplifies bone resorption through osteoclasts and suppresses osteoblast differentiation, which triggers skeletal calcium release [5]. Proinflammatory cytokines, including tumor necrosis factor-alpha, interleukin-1, and interleukin-6, play a central role in this imbalance by linking immune activation with disturbances in mineral metabolism [6]. Inflammation may also interfere with endocrine regulators of calcium homeostasis, particularly parathyroid hormone and vitamin D metabolism [7].

Vitamin D deficiency is highly prevalent in patients with inflammatory rheumatic diseases and reduces intestinal calcium absorption, contributing to secondary hyperparathyroidism and progressive bone loss [8]. This deficiency may also worsen immune dysregulation, creating a reciprocal relationship between calcium imbalance and chronic inflammation [9]. Pharmacologic treatments are widely used in rheumatology and can significantly influence bone and calcium metabolism [10]. Glucocorticoids are among the most commonly used therapies in the management of rheumatic diseases, leading to negative calcium balance through decreased gastrointestinal absorption, increased renal excretion, and the direct inhibition of osteoblast activity [11]. Chronic administration of glucocorticoids is therefore a major contributor to osteoporosis, fragility fractures, and disturbances in calcium homeostasis [12]. Calcium metabolism may also be indirectly influenced by other disease-modifying antirheumatic drugs and biologic agents through the modulation of inflammatory burden, renal function, or gastrointestinal processes [13]. At the same time, widespread use of calcium and vitamin D supplementation, often implemented as a preventive measure, may predispose certain patients to hypercalcemia, nephrolithiasis, and extraskeletal calcification [14].

These potential complications highlight the need for an individualized rather than empirical approach to calcium management in rheumatology [15]. In addition to skeletal effects, calcium disorders may have serious multisystem clinical consequences in patients with rheumatic diseases [16]. Conversely, hypocalcemia may mimic symptoms such as neuromuscular irritability, seizures, paresthesia, or musculoskeletal pain, which can make recognition difficult in clinical settings. Calcium crystal deposition disorders represent an important intersection between mineral dysregulation and inflammatory arthritis [17,18]. Calcium pyrophosphate deposition (CPPD) disease may resemble flares of RA or osteoarthritis and can occur alongside metabolic abnormalities affecting calcium homeostasis [19,20]. Failure to recognize these associations may delay appropriate diagnosis and management [21].

Certain patient groups are particularly vulnerable to calcium disorders in the context of rheumatic diseases [22]. Pediatric-onset rheumatic diseases may impair bone accrual during critical developmental periods, exposing affected individuals to long-term skeletal fragility [23]. Age-related risks, including frailty, sarcopenia, falls, and vascular calcification, may also be aggravated by chronic inflammation and calcium imbalance in older adults [24]. Despite these important implications, calcium disorders are not consistently integrated into routine rheumatologic assessment [25]. Subtle alterations in calcium, phosphate, vitamin D, and parathyroid hormone levels may be overlooked or dismissed during laboratory evaluation [26]. Current clinical guidance largely focuses on osteoporosis screening but provides less emphasis on the broader assessment of calcium dysregulation across rheumatic diseases [27].

A greater understanding of calcium dysregulation in rheumatic diseases is therefore required to improve patient outcomes. Recognizing calcium imbalance as a clinically relevant yet underreported dimension of rheumatic disease may encourage more comprehensive and mechanism-based approaches to patient management.

Objectives of the review

This review aims to provide a structured overview of the spectrum of calcium disorders associated with rheumatic diseases and their underlying pathophysiological mechanisms. It highlights the major clinical manifestations, diagnostic challenges, and metabolic disturbances related to calcium imbalance in these conditions and examines current limitations in recognition and management. Emphasis is placed on the importance of integrating calcium homeostasis assessment into routine rheumatologic evaluation and clinical decision-making. The review synthesizes available evidence to clarify the pathophysiology, clinical implications, and therapeutic considerations of calcium disorders in rheumatic diseases, thereby addressing an underrecognized but clinically relevant aspect of rheumatologic care.

Methodology

Literature Search Strategy

A comprehensive literature search was conducted using three primary electronic databases: PubMed, Scopus, and Web of Science. Searches were performed to identify studies related to calcium metabolism in rheumatologic conditions published between January 2015 and December 2025, capturing both foundational and recent evidence.

Search Terms

The search strategy used combinations of keywords including calcium disorders, hypocalcemia, hypercalcemia, calcium metabolism, rheumatic diseases, autoimmune rheumatic disease, osteoporosis, and other relevant disease-specific terms. Boolean operators were applied to refine the search strategy and improve the retrieval of relevant studies.

Eligibility Criteria

Inclusion criteria: Eligible studies included original research articles, systematic and narrative reviews, clinical trials, observational studies, and case reports addressing calcium abnormalities in adult or pediatric patients with rheumatic diseases.

Exclusion criteria: Non-English publications, animal-only studies, conference abstracts without full-text availability, and articles not directly related to calcium metabolism or its clinical relevance to rheumatic diseases were excluded.

Study Selection Process

Initial screening was performed based on titles and abstracts to determine relevance. Potentially eligible articles then underwent full-text review to determine final inclusion. The screening process was conducted independently by two reviewers, and disagreements regarding study eligibility were resolved through discussion and consensus.

Data Extraction

Data extraction focused on the pathophysiological mechanisms of calcium imbalance, clinical manifestations, diagnostic approaches, and therapeutic strategies. Particular attention was given to the influence of inflammatory activity, pharmacologic therapies, and coexisting metabolic or endocrine conditions on calcium homeostasis. Data extraction was performed independently by two reviewers using a standardized framework to ensure consistency.

Risk of Bias Assessment

The methodological quality and potential risk of bias of the included studies were evaluated independently by two reviewers. The assessment considered study design, methodological rigor, clarity of reported outcomes, and potential sources of bias within individual studies. Any disagreements between reviewers were resolved through discussion.

Data Synthesis

This work represents a narrative review; therefore, no formal quantitative synthesis, such as meta-analysis, pooled estimates, or regression analysis, was conducted. Instead, findings were synthesized qualitatively using a narrative and thematic approach, allowing the integration of evidence across heterogeneous study designs. Extracted data were organized into thematic categories to provide a structured overview of calcium abnormalities in rheumatic diseases, including mechanisms, clinical manifestations, diagnostic considerations, and management strategies.

## Review

Calcium homeostasis in rheumatic diseases

Calcium homeostasis is a well-regulated physiological pathway that maintains skeletal integrity, neuromuscular transmission, vascular function, and intracellular signalling involved in immune cell activation and survival [28]. Rheumatic diseases can disrupt this finely balanced system through chronic inflammation, tissue damage, and metabolic disturbances, making calcium imbalance an underrecognized contributor to overall disease burden [3]. Under normal physiological conditions, calcium balance is maintained through coordinated interactions between the gastrointestinal tract, kidneys, bone, parathyroid hormone, and vitamin D metabolism [7].

Calcium is predominantly stored in bone, and its remodelling is highly sensitive to inflammatory stimuli frequently present in rheumatic diseases [5]. Proinflammatory cytokines enhance osteoclast differentiation and activity, leading to increased bone resorption and the mobilization of calcium into the circulation [6]. At the same time, inflammation suppresses osteoblast activity, impairing bone formation and long-term skeletal mineralization [12]. These processes explain why patients with rheumatic diseases often develop reduced bone mineral density even during the early stages of disease [1].

Beyond skeletal effects, calcium signalling is a key component of T-cell receptor signalling and cytokine production, highlighting the relationship between calcium homeostasis and immune regulation in rheumatic diseases [29]. Calcium imbalance in these conditions generally reflects the consequences of chronic inflammation and immune dysregulation rather than acting as a primary cause of autoimmune disease, although disturbances in calcium homeostasis may further modulate inflammatory responses [10].

The effects are further exacerbated by vitamin D deficiency, which is common in patients with rheumatic diseases and contributes to impaired calcium metabolism and altered immune regulation [8]. Additional complications may arise from kidney disease or medications that alter calcium and phosphate excretion [14]. Furthermore, immobility, aging, nutritional factors, and comorbid endocrine diseases may interact with chronic rheumatic inflammation to increase susceptibility to calcium imbalance [22].

Recognizing calcium dysregulation as an integral component of the pathophysiology of rheumatic diseases is important for understanding downstream complications and improving clinical management strategies [30]. Table 1 summarizes the principal mechanisms contributing to calcium dysregulation in rheumatic diseases.

**Table 1:** Pathophysiological mechanisms underlying calcium dysregulation TNF-α: tumor necrosis factor alpha; IL: interleukin; Ca²⁺: calcium ion

Mechanism	Biological basis	Impact on calcium homeostasis	Clinical implications	Reference
Cytokine-driven bone resorption	TNF-α, IL-1, and IL-6 enhance osteoclastogenesis	Increased skeletal calcium release	Osteoporosis, fracture risk	[6]
Suppressed osteoblast function	Inflammatory inhibition of bone formation	Impaired calcium incorporation into bone	Low bone mineral density	[12]
Vitamin D metabolism impairment	Reduced 1-α hydroxylation during inflammation	Decreased intestinal calcium absorption	Hypocalcemia, secondary hyperparathyroidism	[8]
Secondary hyperparathyroidism	Chronic hypocalcemia and vitamin D deficiency	Increased bone turnover	Skeletal fragility	[1]
Renal tubular dysfunction	Immune-mediated or drug-induced injury	Altered calcium reabsorption/excretion	Nephrolithiasis, hypercalcemia	[14]
Immune cell calcium signalling	Abnormal intracellular Ca²⁺ flux	Sustained immune activation	Persistent inflammation	[29]

Pathophysiological mechanisms of calcium dysregulation

The complex interplay between chronic inflammation, immune activation, endocrine disruption, and organ-specific involvement forms the basis of calcium dysregulation in rheumatic diseases [31]. Persistent systemic inflammation is one of the major mediators of disrupted calcium homeostasis and primarily affects bone remodelling and mineral metabolism [5]. Proinflammatory cytokines such as tumor necrosis factor-alpha, interleukin-1, and interleukin-6 stimulate osteoclastogenesis and suppress osteoblast differentiation, resulting in increased bone resorption and the mobilization of calcium into the circulation [6]. These processes contribute to progressive skeletal fragility and disturbances in serum calcium levels over time [12].

Inflammation also interferes with the hormonal regulation of calcium homeostasis, particularly parathyroid hormone and vitamin D metabolism [7]. Cytokine-mediated inhibition of vitamin D activation reduces intestinal calcium absorption, exposing patients to hypocalcemia and secondary hyperparathyroidism [8]. In response, elevated parathyroid hormone levels further stimulate bone turnover, creating a cycle of bone loss and mineral imbalance [1]. This process is particularly evident in patients with high disease activity or persistent inflammatory burden [3].

Renal involvement is another significant contributor to calcium dysregulation in rheumatic diseases [14]. Immune-mediated damage to glomeruli or renal tubules can impair calcium reabsorption and phosphate handling [18]. Prolonged inflammation may also alter the renal expression of calcium transport proteins, leading to changes in urinary calcium excretion [32]. These alterations predispose patients to nephrolithiasis and renal calcification, particularly in individuals receiving calcium supplementation or long-term glucocorticoid therapy [9].

Disturbances in intracellular calcium signalling may also influence immune cell behavior [29]. Abnormal calcium influx into immune cells can alter T-cell activation, cytokine release, and apoptosis, thereby sustaining chronic inflammation [10]. This reflects a bidirectional relationship in which calcium imbalance both results from and contributes to immune dysregulation in rheumatic diseases [28].

**Figure 1:**
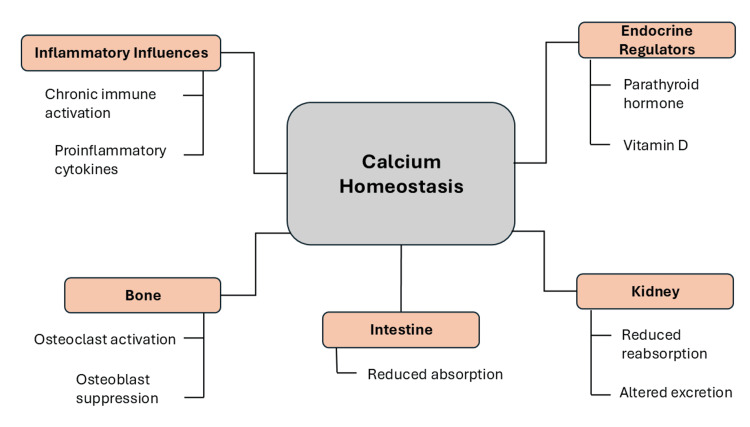
Pathophysiological mechanisms of calcium dysregulation in rheumatic diseases Image created by the authors using Microsoft PowerPoint (Microsoft Corporation, Redmond, Washington, United States)

Overall, these mechanisms indicate that calcium disorders in rheumatic diseases represent complex systemic disturbances rather than isolated metabolic abnormalities [30]. Recognizing these interconnected mechanisms is essential for identifying at-risk patients and developing strategies to prevent long-term complications [4]. The alterations in physiological calcium regulation in rheumatic diseases are illustrated in Figure 1, which demonstrates how immune-mediated inflammation disrupts normal calcium homeostasis.

Impact of rheumatic disease-related therapies on calcium balance

Medications applied in the management of rheumatic ailments produce tremendous and often intricate effects on the calcium homeostasis, which may result in acute and chronic metabolic issues [33]. Among them, glucocorticoids are still the strongest medications that affect the calcium balance due to their popularity and skeletal toxicity, which are already well-known [11]. Glucocorticoids reduce small intestinal calcium absorption, increase renal calcium excretion, and directly inhibit osteoblast differentiation and increase osteocyte apoptosis, which leads to negative calcium balance and accelerated bone loss [12]. They may be manifested at the start of treatment and escalate with the dose and the time of exposure [5]. The conventional disease-modifying antirheumatic drugs have the potential to indirectly control the calcium metabolism by controlling the inflammatory load and organ processes [13]. The restoration of bone remodelling balance, as well as the improvement of calcium metabolism, may result partly through the effective mitigation of systemic inflammation, and the inability to manage the illness continues to endanger the formation of a cytokine-driven bone resorption [6]. The actions of methotrexate and other antimetabolites on bone path in certain settings are also connected to the action of disease severity, as well as in the utilization of other glucocorticoids [1].

**Figure 2:**
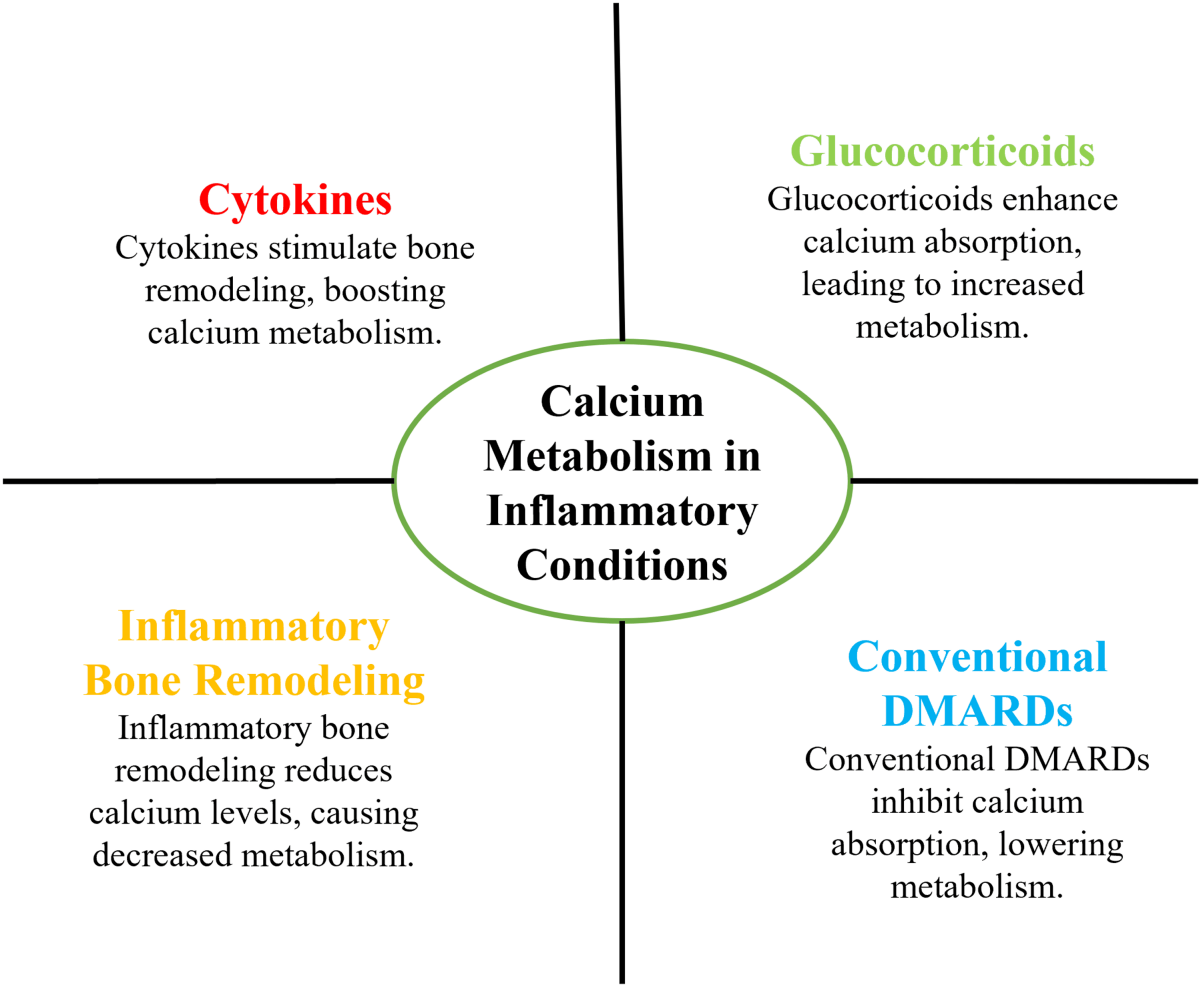
Interaction between inflammation, therapy, and calcium metabolism DMARDs: disease-modifying antirheumatic drugs Image created by the authors using Microsoft PowerPoint (Microsoft Corporation, Redmond, Washington, United States)

Biologic and targeted synthetic agents complicated the calcium regulation even more [29]. Such treatments have the potential to suppress osteoclasts and inflammatory effects and inhibit calcium mobilization through the selective inhibition of major inflammatory pathways [10]. However, it is not clear how they are affected in the long term in mineral metabolism, particularly concerning renal calcium and in response to supplementation interventions [18]. There has been inconsistent evidence of the effect of interleukin-6 blockers like tumor necrosis factor on the bone density and calcium markers in different cohorts of patients [7], but calcium and vitamin D supplement is commonly prescribed to reverse the influence of bone loss caused by treatment, although inappropriate use in susceptible patients may result in hypercalcemia, nephrolithiasis, and extraskeletal calcification [14]. These side effects can pose a particular risk to patients with renal defects or sarcoid-like reactions or those with the occurrence of altered vitamin D metabolism [9]. This is increasingly being proven to suggest that excess dosing of calcium supplementation may cause vascular calcification, which increases cardiovascular risk that is already high in rheumatic diseases [22]. The results emphasize the necessity of individualized treatment methods that cannot merely manage this disease but also closely monitor the calcium metabolism [30]. The decision-making process of the treatment process should include calcium assessment to minimize the effects, which are directed toward an iatrogenic nature, and enhance the long-term results of the patients with rheumatic diseases [34]. Figure 2 indicates the interaction between disease activity and pharmacologic therapy and calcium metabolism.

Spectrum of calcium disorders in rheumatic diseases

Overview

Calcium disorders represent a heterogeneous spectrum of conditions in rheumatic diseases that reflect underlying inflammatory processes, organ involvement, and treatment effects [35]. These abnormalities range from asymptomatic biochemical imbalances to clinically significant conditions associated with substantial morbidity, underscoring their relevance to routine rheumatologic practice [28]. The most familiar manifestations include hypocalcemia and hypercalcemia, while other important but less frequently recognized entities include secondary hyperparathyroidism and calcium crystal-related disorders [7].

Hypocalcemia in Rheumatic Diseases

Hypocalcemia in rheumatic diseases is most commonly associated with vitamin D deficiency, intestinal malabsorption, renal failure, and chronic inflammation [8]. It is particularly prevalent in patients with high disease activity or those exposed to long-term glucocorticoid therapy, since inflammation disrupts vitamin D activity and parathyroid hormone responsiveness [11]. Neuromuscular irritability, paresthesia, muscle cramps, or refractory musculoskeletal pain may be clinical manifestations of hypocalcemia, which may be mistakenly attributed to active inflammatory disease [18]. Even subclinical hypocalcemia may contribute to long-term bone demineralization and increased fracture risk [12].

Hypercalcemia in Rheumatic Diseases

Hypercalcemia, although less common, has important diagnostic and therapeutic implications in patients with rheumatic diseases [36]. It may occur due to excessive calcium or vitamin D intake, granulomatous inflammation with extra-renal activation of vitamin D, prolonged immobilization, or underlying malignancy [9]. Hypercalcemia may present insidiously with symptoms such as fatigue, cognitive impairment, polyuria, and renal dysfunction, and it may therefore be detected late in patients with complex multisystem disease [17]. In severe cases, hypercalcemia can lead to cardiac arrhythmias or renal failure [14].

Secondary Hyperparathyroidism

Secondary hyperparathyroidism represents an adaptive response to chronic hypocalcemia and vitamin D deficiency and is frequently observed in inflammatory rheumatic diseases [1]. Elevated parathyroid hormone levels increase bone turnover and contribute to progressive skeletal fragility in patients with long-standing disease [5].

Calcium Crystal Deposition Disorders

Calcium crystal disorders, particularly CPPD disease, represent local disturbances of mineral metabolism that manifest as episodic inflammatory arthritis [10]. Multiple calcium abnormalities may coexist in the same patient because calcium dysregulation in rheumatic diseases is multifactorial [33].

Clinical Implications

Recognition of this spectrum of calcium abnormalities is essential for the accurate diagnosis, targeted management, and prevention of downstream complications [30]. Appropriate evaluation and management of calcium disorders may therefore improve both metabolic and rheumatologic outcomes in affected patients [37]. Table 2 summarizes the spectrum, clinical presentation, and diagnostic approaches to calcium disorders in rheumatology.

**Table 2:** Spectrum and diagnosis of calcium disorders in rheumatic diseases CPPD: calcium pyrophosphate deposition; PTH: parathyroid hormone; Ca: calcium

Disorder	Common associations	Key clinical features	Diagnostic approach	Reference
Hypocalcemia	Vitamin D deficiency, renal disease	Muscle cramps, paresthesia, seizures	Corrected calcium, PTH, and vitamin D	[18]
Hypercalcemia	Excess supplementation, granulomatous disease	Fatigue, polyuria, renal dysfunction	Serum calcium, PTH, and renal function	[36]
Secondary hyperparathyroidism	Chronic inflammation, low vitamin D	Bone pain, fractures	Elevated PTH with low/normal Ca	[5]
CPPD disease	Aging, metabolic disorders	Acute or chronic inflammatory arthritis	Synovial fluid crystal analysis	[20]
Basic calcium phosphate deposition	Degenerative/inflammatory joints	Tendinitis, bursitis	Imaging, exclusion diagnosis	[37]
Extraskeletal calcification	Long-standing inflammation	Vascular or valvular disease	Imaging modalities	[30]

Calcium pyrophosphate and other crystal deposition diseases

Calcium crystal deposition diseases represent a distinct interface between disturbed mineral metabolism and inflammatory arthritis and occupy an important yet often underrecognized place in rheumatology [38]. The most common and clinically significant of these conditions is CPPD disease, which may clinically resemble RA, osteoarthritis, or acute crystal-induced synovitis [19]. CPPD disease is characterized by the deposition of calcium pyrophosphate crystals in articular and periarticular tissues, leading to acute or chronic inflammatory manifestations [10].

The pathophysiology of CPPD disease is complex and is closely related to disturbances in calcium and phosphate homeostasis [7]. Crystal formation and deposition are associated with aging, metabolic disorders such as hyperparathyroidism and hypomagnesemia, chronic kidney disease, and alterations in cartilage metabolism [1]. Inflammatory rheumatic diseases may further predispose patients to CPPD disease through persistent joint damage, cartilage degeneration, and systemic metabolic disturbances [5]. These overlapping mechanisms often complicate the clinical distinction between CPPD disease and primary inflammatory arthritides [28].

Clinical manifestations of CPPD disease are highly variable, ranging from asymptomatic chondrocalcinosis to acute pseudogout attacks and chronic inflammatory arthritis resembling RA [20]. Acute attacks typically present with sudden-onset joint pain, swelling, and erythema and most frequently affect the knees, wrists, and ankles [18].

Chronic CPPD disease may lead to persistent synovitis, progressive joint destruction, and functional impairment, particularly in older patients with coexisting rheumatic diseases [22]. Diagnosis remains challenging because radiographic chondrocalcinosis may lack sensitivity and can be absent during early disease stages [14]. The diagnostic gold standard is synovial fluid analysis demonstrating positively birefringent rhomboid-shaped crystals, although imaging modalities such as ultrasound have improved detection rates [11]. Misdiagnosis may result in the inappropriate escalation or reduction of immunosuppressive therapy and failure to address underlying metabolic abnormalities [30].

Other calcium crystal disorders, including basic calcium phosphate deposition, may also trigger inflammatory syndromes and periarticular calcification in rheumatic diseases [39]. These conditions are associated with tendinopathies, bursitis, and destructive arthropathies and further contribute to the clinical burden of calcium dysregulation [9]. Recognition of crystal-related disorders is therefore essential for accurate diagnosis, targeted treatment, and avoidance of unnecessary immunosuppressive therapy [37].

**Figure 3:**
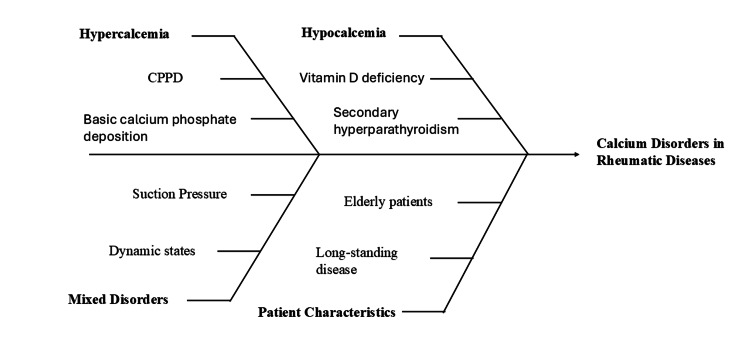
Calcium disorders in rheumatology CPPD: calcium pyrophosphate deposition Image created by the authors using Microsoft PowerPoint (Microsoft Corporation, Redmond, Washington, United States)

The role of mineral imbalance in the development and progression of rheumatic diseases has long been recognized through the study of calcium crystal disorders [33]. Integrating metabolic evaluation into the assessment of crystal-induced arthritis may improve diagnostic accuracy and support more personalized treatment strategies [40]. The range of calcium-related abnormalities encountered in rheumatologic practice is illustrated in Figure 3.

Clinical manifestations and multisystem implications

The clinical manifestations of calcium disorders in rheumatic diseases are highly diverse and extend beyond the skeletal system, reflecting the systemic importance of calcium homeostasis [41]. The most well-recognized consequences involve skeletal complications, as chronic calcium imbalance in patients with inflammatory rheumatic diseases may lead to osteoporosis, reduced bone mineral density, and an increased risk of fractures [12]. These skeletal effects are primarily driven by inflammation-induced bone resorption, prolonged glucocorticoid exposure, and secondary hyperparathyroidism, which frequently coexist in this patient population [5].

Neuromuscular symptoms represent another important but often underrecognized manifestation of calcium imbalance [18]. Hypocalcemia may present with muscle cramps, tetany, paresthesia, seizures, or nonspecific musculoskeletal pain, which can be mistaken for manifestations of active inflammatory disease or fibromyalgia [9]. Conversely, hypercalcemia may cause proximal muscle weakness, fatigue, and neurocognitive impairment, complicating clinical evaluation and affecting quality of life in patients attending rheumatology clinics [17]. These overlapping clinical features may contribute to the delayed recognition and underdiagnosis of calcium disorders [28].

Renal involvement represents another significant clinical consequence of calcium imbalance in rheumatic diseases [14]. Well-recognized complications include hypercalciuria and nephrolithiasis, particularly in patients receiving calcium or vitamin D supplementation or those with underlying renal disease [33]. Progressive renal calcification may further disrupt calcium and phosphate homeostasis, creating a cycle of metabolic disturbance [1]. In severe cases of hypercalcemia, acute kidney injury may also occur and contribute to additional clinical morbidity [36].

Cardiovascular manifestations represent another important downstream effect of disrupted calcium metabolism [42]. Extraskeletal calcification, including vascular and valvular calcification, may contribute to accelerated atherosclerosis and increased cardiovascular risk, particularly in patients with chronic inflammatory rheumatic diseases [22]. Emerging evidence also suggests that excessive calcium supplementation may promote these processes, highlighting the importance of careful risk-benefit assessment when prescribing supplementation [7].

Calcium disorders may also produce gastrointestinal symptoms. Hypercalcemia is associated with constipation, nausea, and abdominal pain, whereas chronic hypocalcemia may impair nutritional status and intestinal absorption [8]. These multisystem manifestations may be particularly pronounced in children and older adults, where calcium imbalance may contribute to impaired growth, frailty, and reduced functional reserve [23].

The multisystem nature of calcium disorders underscores their clinical significance and may complicate disease assessment in rheumatologic practice [30]. Systematic recognition of these manifestations is essential for early diagnosis, the prevention of complications, and the optimization of comprehensive patient care [43].

Special populations

Certain patient populations are particularly vulnerable to calcium disorders in the context of rheumatic diseases due to physiological, developmental, and comorbidity-related factors that increase susceptibility to metabolic disturbances [44]. Children with rheumatic diseases represent an important high-risk group in whom calcium imbalance may have long-term consequences [23]. Chronic inflammation during critical stages of growth can interfere with peak bone mass acquisition, while glucocorticoid exposure and reduced physical activity further impair skeletal mineralization [12]. Vitamin D deficiency with secondary hyperparathyroidism is also common in this population and may predispose affected individuals to hypocalcemia and increased fracture risk later in life [8].

Older adults with rheumatic diseases constitute another population at increased risk of calcium dysregulation [22]. Age-related physiological changes, including reduced renal function, decreased intestinal calcium absorption, and diminished vitamin D synthesis, may amplify the effects of chronic inflammation and polypharmacy [7]. These patients are particularly susceptible to osteoporosis, supplementation-related hypercalcemia, and calcium crystal deposition disorders such as CPPD disease, which may clinically resemble degenerative or inflammatory arthritis [20]. Calcium imbalance may also contribute to frailty, sarcopenia, and increased risk of falls, thereby increasing morbidity and loss of independence in this population [24].

Patients with long-standing or severe rheumatic disease are also more prone to developing calcium disorders [5]. Cumulative glucocorticoid exposure, persistent inflammatory activity, and involvement of multiple organ systems, including renal and gastrointestinal comorbidities, create a complex metabolic environment that predisposes patients to both hypocalcemic and hypercalcemic states [14]. In these individuals, calcium disturbances may fluctuate over time, making recognition and management more challenging [33].

Another vulnerable group includes patients with coexisting endocrine or metabolic disorders [1]. Conditions such as thyroid disease, diabetes mellitus, and chronic kidney disease may further disrupt calcium and phosphate homeostasis when combined with inflammatory rheumatic disease [9]. These interactions may increase the risk of complications such as nephrolithiasis, vascular calcification, and accelerated bone loss [36].

Women, particularly postmenopausal patients with inflammatory rheumatic diseases, represent another high-risk population due to estrogen deficiency, inflammatory bone loss, and calcium imbalance [12]. Pregnancy and lactation also impose additional physiological demands on calcium metabolism, requiring careful monitoring in women with active rheumatic disease [28].

Recognition of these vulnerable populations is essential for effective risk stratification and the implementation of individualized management strategies [30]. Targeted monitoring of calcium metabolism and personalized interventions may help improve outcomes and reduce long-term complications in diverse rheumatologic patient groups [45].

Diagnostic considerations and laboratory assessment

Accurate and context-specific diagnosis of calcium disorders in rheumatic diseases can be challenging because biochemical abnormalities are often influenced by inflammatory, renal, and treatment-related confounding factors [46]. The main screening test is measurement of serum total calcium; however, the results must be interpreted in relation to serum albumin levels, acid-base status, and coexisting diseases, which are frequently altered in patients with rheumatic conditions [7]. These factors may lead to the misclassification of calcium status and inappropriate clinical decisions if not properly corrected or considered [28].

Measurement of ionized calcium (Ca²⁺) provides a more accurate estimate of biologically active calcium, particularly in critically ill patients or in those who are markedly hypoalbuminemic [14]. Despite its diagnostic value, routine measurement of ionized calcium is often limited by restricted availability, pre-analytical variability, and technical requirements, which means that interpretation of total calcium values remains common in clinical practice [9]. Repeated measurements may also be required, as calcium levels may fluctuate with disease activity, medication changes, or the presence of comorbid conditions [33].

Comprehensive evaluation of calcium disorders extends beyond the measurement of serum calcium alone and should include the assessment of phosphate, magnesium, parathyroid hormone, and vitamin D levels [1]. Measurement of 25-hydroxyvitamin D is particularly important for identifying deficiency states that contribute to secondary hyperparathyroidism and hypocalcemia in rheumatic diseases [8]. Elevated parathyroid hormone levels in the presence of normal or low calcium should prompt the consideration of secondary hyperparathyroidism related to chronic inflammation or vitamin D deficiency rather than primary endocrine disease [5].

Assessment of renal function is also essential, as impaired glomerular or tubular function can significantly alter calcium and phosphate regulation [18]. Measurement of urinary calcium excretion may help differentiate the causes of hypercalcemia and identify patients at risk of nephrolithiasis, particularly those receiving calcium supplementation [36].

Imaging modalities such as dual-energy X-ray absorptiometry, conventional radiography, and ultrasound may support the evaluation of skeletal involvement and the detection of crystal deposition disorders [11]. However, diagnostic challenges remain because the clinical manifestations of calcium disorders may mimic symptoms of active rheumatic disease or adverse effects of medications used in treatment [17]. Inflammatory markers may further obscure underlying metabolic abnormalities, complicating diagnosis [10].

A multidisciplinary approach involving rheumatologists, endocrinologists, and nephrologists may improve diagnostic accuracy and optimize patient outcomes [30]. Incorporating structured laboratory assessment of calcium metabolism into routine rheumatologic care may facilitate the early detection and management of calcium disorders [45]. A proactive diagnostic strategy can help prevent complications, guide personalized treatment, and improve long-term clinical outcomes in patients with rheumatic diseases [47].

Therapeutic and management strategies

Management of calcium disorders in rheumatic diseases requires an individualized approach that addresses underlying disease activity, metabolic abnormalities, and treatment-related risks [48]. Optimal management begins with effective control of the underlying inflammatory disease, as successful suppression of systemic inflammation may partially restore bone remodelling and calcium metabolism [6]. Disease-modifying antirheumatic drugs and biologic therapies can reduce cytokine-mediated bone resorption and limit progressive disturbances in calcium homeostasis [10].

Calcium and vitamin D supplementation remain important components in the prevention and treatment of osteoporosis and hypocalcemia in patients with rheumatic diseases, but supplementation should be guided by individual risk assessment [14]. Before initiating supplementation, baseline evaluation of serum calcium, vitamin D status, renal function, and parathyroid hormone levels is recommended to avoid overtreatment and potential adverse effects [1]. In patients with confirmed deficiency, vitamin D replacement improves intestinal calcium absorption and may provide additional immunomodulatory benefits [8]. However, supplementation should be used cautiously, particularly in patients with renal impairment, granulomatous disease, or a history of nephrolithiasis [36].

Calcium imbalance associated with glucocorticoid therapy requires preventive management strategies [11]. Use of the lowest effective glucocorticoid dose, early introduction of steroid-sparing agents, and regular reassessment of fracture risk are essential components of management [12]. Bone-targeted therapies such as bisphosphonates may be recommended for high-risk patients to prevent bone loss and maintain calcium balance [5].

Management of hypercalcemia focuses on identifying and correcting the underlying cause, discontinuing unnecessary calcium supplementation, and ensuring adequate hydration to increase renal calcium excretion [17]. In severe cases, pharmacologic interventions may be required to rapidly reduce serum calcium levels and prevent organ dysfunction [9]. In calcium crystal deposition disorders, treatment primarily involves control of acute inflammation, correction of underlying metabolic abnormalities, and avoidance of unnecessary immunosuppressive therapy [20].

Nonpharmacologic interventions also play a crucial role in maintaining calcium balance and skeletal health [22]. Adequate nutritional intake, weight-bearing physical activity, fall-prevention strategies, and patient education contribute to improved long-term musculoskeletal outcomes [24]. Regular monitoring of calcium-related parameters is recommended, as calcium balance may fluctuate with disease progression, aging, and therapeutic interventions [33].

Effective management of calcium disorders in rheumatic diseases requires a multidisciplinary approach involving rheumatologists, endocrinologists, and nephrologists [30]. Integrating metabolic assessment into routine rheumatologic care may reduce complications, improve quality of life, and support long-term disease management [49]. Table 3 summarizes the management strategies for calcium disorders in the context of rheumatic diseases.

**Table 3:** Therapeutic strategies and clinical application Ca: calcium; Vit D: vitamin D

Clinical context	Management strategy	Key considerations	Expected outcome	Reference
Active inflammatory disease	Aggressive disease control	Reduce cytokine-driven bone loss	Improved calcium balance	[6]
Vit D deficiency	Targeted Vit D replacement	Monitor calcium and renal function	Normalized absorption	[8]
Glucocorticoid therapy	Dose minimization, steroid-sparing agents	Early fracture risk assessment	Bone protection	[11]
Osteoporosis risk	Anti-resorptive therapy	Individualized risk stratification	Reduced fractures	[12]
Hypercalcemia	Stop supplements, hydration	Identify the underlying cause	Ca normalization	[17]
High-risk populations	Individualized monitoring	Age, renal disease, comorbidities	Complication prevention	[45]

Bridging mechanistic insights to clinical application

There is a need to translate mechanistic information on calcium dysregulation to the actual rheumatologic practice so that morbidity, which is preventable, can be reduced and the long-term outcomes can be improved [50]. The understanding of the inflammation-induced bone remodelling, endocrine perturbation, and immune-calcium signalling pathophysiology has been developed to contribute to the explanation of the fact that calcium disorders are not incidental but a part and parcel of the biology of the disease [31]. Knowledge of this paradigm shift will make clinicians incorporate the measurement of calcium in the general assessment of a disease, rather than a screening for osteoporosis [30]. Clinical practice involves a crucial step of risk identification of patients [46]. High amounts of inflammatory burden, years of chronic glucocorticoids, renal disease, and vitamin D deficiency are the signs, which mean that patients are to be provided with an organized metabolic assessment, although there are no evident symptoms [8]. Risk stratification allows clinicians to estimate the complications in relation to calcium and change the frequency of the monitoring and preventive care depending on the estimation [22]. These are proactive interventions that are consistent with the broader trend as far as personalized medicine is concerned in rheumatology [48].

Mechanistic understandings also inform the therapeutic decision-making [33]. Being aware of the impact of some treatments on the calcium balance assists in making rational decisions in the glucocorticoid-sparing regimen, cautious supplementation, and early initiation of bone-protective interventions [11]. The iatrogenic harm could be reduced with the help of metabolic considerations in the treatment planning, and the disease management could be stable [12]. Calcium crystal pathophysiology may be used in the prevention of misdiagnosis and unsuitable intensification of immunosuppression [20]. Multidisciplinary collaboration is the key target of successful transfer of knowledge to practice [14]. The primary care providers, endocrinologists, nephrologists, rheumatologists, and others work hand-in-hand to enable holistic evaluation of complex calcium conditions and continuity of care [1]. Patient education is also essential to ensure that individuals understand the reasons for monitoring, supplementation, and recommended lifestyle modifications [24].

Regarding the system, the implementation of standardized calcium testing programs in the rheumatology clinics can prove helpful in the aspects of the rates of detection and adherence to the care [45]. Combining lab prompts/decision-support systems into the clinical practice can help bridge the gaps in the evidence for practitioners [47]. The plans are particularly appropriate in resource-limited settings where metabolic challenges are commonly not well-detected [28]. The clinical to mechanistic translation of the knowledge of calcium disorders will assist in targeting it as a clinical burden of disease rather than as the inevitable comorbid condition [41]. Integrating pathophysiological knowledge and clinical practice, rheumatology care will be designed towards a more holistic and preventive methodology that has a high implication for patient outcomes [50].

Limitations and future directions

This review has a few limitations that should be taken into consideration. It is a narrative review and is dependent on the published literature available, which was not conducted based on a systematic review or a meta-analytic response, which may create bias in selection. The heterogeneity of the studies in terms of disease populations and procedures for defining calcium disorders and diagnostic levels, and outcome measures, limits the direct comparison and generalization of the results. Various studies that exist are observational or cross-sectional, which restrict causation. There is little data about the long-term clinical outcomes of calcium disorders and the effects of more recent targeted therapy on calcium homeostasis. The subclinical calcium abnormalities will probably be underreported due to the infrequency with which screening is performed in the rheumatology practice.

It has been suggested that future longitudinal studies be done to determine the prevalence, natural history, and prognostic implications of calcium disorders in different rheumatic diseases. Standardized diagnostic algorithms should also exist, and implementation of standard reporting of the calcium-related outcomes should also exist, something that would facilitate comparability between the studies. New therapeutic targets can be established through immune-calcium signalling (mechanistic) research. Interventional trials should consider individualized strategies of calcium and vitamin D, particularly in the high-risk groups. The possible incorporation of calcium assessment in clinical practice may ultimately result in a more integrated approach to care and the lack of possibility to enhance the outcome of rheumatology in the long term.

## Conclusions

Calcium disorders represent a pervasive yet underrecognized dimension of rheumatic diseases, intersecting inflammation, immunity, metabolism, and long-term organ outcomes. Across diverse rheumatic conditions, disruptions in calcium homeostasis arise from disease activity, therapeutic exposures, and comorbid metabolic factors, collectively shaping skeletal and extraskeletal morbidity. This review highlights that calcium imbalance is not merely a secondary complication but an integral component of rheumatic disease biology with meaningful clinical consequences. Failure to recognize these disturbances may obscure diagnosis, confound symptom assessment, and amplify preventable complications. Integrating calcium evaluation into routine rheumatologic care offers an opportunity to move toward more holistic, preventive, and patient-centered management. Mechanistic insights can inform rational therapeutic decisions, individualized supplementation strategies, and timely intervention for high-risk populations. A multidisciplinary approach that bridges immunology, endocrinology, nephrology, and rheumatology is essential to translate knowledge into practice. By reframing calcium disorders as modifiable contributors to disease burden rather than incidental findings, clinicians can enhance long-term musculoskeletal health, reduce systemic complications, and improve overall outcomes. This perspective supports comprehensive, integrated, and sustainable rheumatologic care.
